# Contrasting SARS-CoV-2 RNA copies and clinical symptoms in a large cohort of Colombian patients during the first wave of the COVID-19 pandemic

**DOI:** 10.1186/s12941-021-00445-8

**Published:** 2021-05-24

**Authors:** Santiago A. Quiroga, Carolina Hernández, Sergio Castañeda, Paula Jimenez, Laura Vega, Marcela Gomez, David Martinez, Nathalia Ballesteros, Marina Muñoz, Claudia Cifuentes, Nathalia Sierra, Carolina Flórez, Alberto Paniz-Mondolfi, Juan David Ramírez

**Affiliations:** 1 Centro de Investigaciones en Microbiología y Biotecnología-UR (CIMBIUR), Facultad de Ciencias Naturales, Universidad del Rosario, Bogotá, Colombia; 2 Escuela de Medicina y Ciencias de la Salud, Universidad del Rosario, Bogotá, Colombia; 3 Laboratorio de Salud Pública, Dirección de Salud Pública, Secretaria de Salud de Cundinamarca, Bogotá, Colombia; 4 Instituto Nacional de Salud, Bogotá, Colombia; 5 Instituto de Investigaciones Biomédicas IDB/Incubadora Venezolana de la Ciencia, Barquisimeto, Venezuela; 6 Icahn School of Medicine at Mount Sinai, New York, USA

**Keywords:** SARS-CoV-2, COVID-19, RT-PCR, Ct, Clinical symptoms, Comorbidities

## Abstract

**Background:**

There is limited and controverting evidence looking at possible associations of severe acute respiratory syndrome coronavirus 2 (SARS-CoV-2) RNA copies and patient variables in large cohorts of symptomatic and asymptomatic patients.

**Methods:**

We studied 2275 symptomatic and asymptomatic patients from Colombia with coronavirus disease 2019 (COVID-19) and analyzed the associations between RT-PCR cycle threshold (Ct) value with gender, age, comorbidities, symptomatology, and disease severity.

**Results:**

15.4 % of the samples (n = 428) reported at least one comorbidity. There were 2011 symptomatic cases (72.4 %), being the most common reported symptom cough (57.2 %, n = 1586). Respiratory distress was present in 21.4 % of patients (n = 595), and 435 patients (15.6 %) required hospital admission. We observed that patients with no prior medical history harbored higher RNA copies than patients with comorbidities (p = 0.02). No significant differences in RNA copies were observed between symptomatic and asymptomatic patients (p = 0.82). Strong correlations were detected between Ct values and the presence of odynophagia (p = 0.03), diarrhea (p = 0.04), and headache (p = 0.0008). An inverse association was found between RNA copy number and markers of disease severity, namely, respiratory distress (P < 0.0001) and hospitalization requirement (P < 0.0001).

**Conclusions:**

SARS-CoV-2 RT-PCR cycle thresholds reveal strong associations with a prior medical history, specific symptomatology, and disease severity markers. Further research controlling potential confounding variables needs to be conducted to evaluate the nature and usefulness of these associations in managing COVID-19 patients.

**Supplementary Information:**

The online version contains supplementary material available at 10.1186/s12941-021-00445-8.

## Background

In December of 2019, following a cluster of pneumonia cases in Wuhan, China, a novel beta coronavirus was discovered [1] As of March 28, 2021, the now termed severe acute respiratory syndrome coronavirus 2 (SARS-CoV-2) has spread over 220 countries, causing approximately 100 million confirmed infections and more than 2.4 million associated deaths [2]. In Colombia, the coronavirus disease 2019 (COVID-19) has led to more than 2.1 million confirmed cases and 55 thousand reported deaths [3].

A key aspect in response to the COVID-19 pandemic has been the early detection and prompt isolation of infected patients [4]. For this purpose, real-time reverse transcriptase-polymerase chain reaction (RT-PCR) has been regarded as the gold standard diagnostic test [5], with an estimated 70 and 95 % sensitivity and specificity, respectively [6] RT-PCR reveals the broadest range of detection: becoming positive days before symptom onset and detecting viral nucleic acids up to several weeks in infected patients [7–9]. However, it is essential to clarify that test performance may differ due to the inherent variability between assays, extraction method/instrument combination, targeted regions of the viral genome, and clinical matrix tested. This makes standardization of Ct values and comparison across different platforms a challenging task.

One of the benefits of using real-time RT-PCR in diagnosing COVID-19 patients is generating the Cycle Threshold (Ct). This value represents the amplification cycle at which viral nucleic acids reach a detectable level [10]. It is considered a proxy marker of viral load: a higher Ct value implies that more replication cycles are needed to detect RNA. A lower Ct value suggests a higher number of viral copies are present in the sample taking fewer cycles to reach a detectable level [7, 10]. However, it must be noted that the Ct value is not a direct marker of viral load [11], and thus, we will be using the term “RNA copies” instead of viral load. By consensus, a test is considered positive when the Ct value is lower than 40 and is observed a typical S-shape amplification curve [7]. The Ct remains the most accessible way to indirectly assess RNA copies in COVID-19 patients, although no sound clinical studies validate the use of Ct values to guide COVID-19 management. Because of this, since the very start of the COVID-19 pandemic, the scientific community has invested many efforts in studying the potential correlations and applicability of Ct values in the clinical context and how this may affect different parameters in COVID-19 patients (12–22).

For instance, by using Vero cell culture models, some authors have analyzed how Ct value samples collected from RT-PCR positive patients still retain infectivity [12, 13]. Although the specific cut-off point varies according to each assay and target gene used, the data derived from these studies highlights that not all positive RT-PCR tests reflect the presence of viable viral particles [7, 12, 13]. This, in turn, has helped tailor patient isolation recommendations and eliminate the need for follow-up RT-PCR in most cases [14]. In contrast, other studies have analyzed Ct values’ distribution among symptomatic and asymptomatic patients [8, 15–20]. Mainly, Zou et al. were among the first authors to demonstrate that asymptomatic patients could have RNA copies comparable to those of symptomatic cases—highlighting the importance of community isolation measures to prevent the spread of SARS-CoV-2 [15].

Ct values have also been used to assess viral load dynamics of SARS-CoV-2 in both symptomatic and asymptomatic cases [9, 21, 22]. Most of these studies report that RNA copies and infectivity start rapidly rising 0-to-6 days before the onset of symptoms, reaching a peak near symptom onset, to quickly decline within seven days [9, 22]. These findings had shed light on the role of asymptomatic and presymptomatic patients in the spread of SARS-CoV-2 and the importance of contact tracing and isolation precautions for close contacts [14]. Other studies have examined the potential role of the Ct value as a prognostic marker in COVID-19 to identify patients at high risk for severe disease [23–31]. However, available data on this subject is contradictory; with some studies showing an independent association between RNA copies and risk of intubation or death [23–29]. The latter has been supported by studies that report significantly lower RNA copies in patients with severe disease [30, 32, 33]. However, other international cohorts have not detected such trend [21] and even report observing significantly lower RNA copies in patients with severe disease [34–36].

The tendency of these studies to concentrate on symptomatic and hospitalized patients, along with the small sample size present in most cohorts, has made interpretation of data difficult. Also, the lack of large South American cohorts and the circulation of the Variant of Concern P.1 also poses challenges in the extrapolation of findings. Herein we studied the Ct values of symptomatic and asymptomatic patients with COVID-19 and their potential associations with age, gender, comorbidities, symptomatology, and disease severity in a sample of 2275 patients in Cundinamarca (Central Colombia).

## Methods

### Data collection

From March 1 through September 13, 2020 (first epidemic wave in Colombia), a total of 27.860 nasopharyngeal samples were collected from patients from the Cundinamarca department. Physicians obtained the samples during the evaluation of patients with suspected COVID-19 across various inpatient and outpatient settings across the department of Cundinamarca (Samples from Bogotá were not included in this study) in central Colombia (Additional file 1: Figure S1).

Patient data were collected through a national-level standardized form (Additional file 2: Figure S2) recommended by the Colombian ministry of health. The form explicitly asked providers to fill in the following patient information: date, name, age, gender, department, symptoms, comorbidities, and hospital admission. Regarding symptomatology, physicians were explicitly required to state the presence or absence of cough, fever, odynophagia, fatigue, rhinorrhea, headache, conjunctivitis, diarrhea, and respiratory distress. Regarding prior medical history, medical professionals were also required to state the presence or absence of asthma, Chronic Obstructive Pulmonary Disease (COPD), Diabetes, Infection by Human Immunodeficiency Virus (HIV), Cardiac disease, Cancer, Malnutrition, Obesity, Renal insufficiency, use of immunosuppressive drugs, Tuberculosis, and Smoking status. The form also required additional data like ethnicity, telephone number, and previous antibiotic use.

### Ethics statement

The Colombian National Institute of Health (INS) is designated as the reference laboratory in Colombia. When a public health emergency occurs, the INS is authorized under national law 9-1979, decrees 786–1990 and 2323− 2006, to use biospecimens and associated epidemiological information without informed consent, including the anonymous disclosure of results. This study was performed following the Declaration of Helsinki and its later amendments, and all patient data was anonymized to minimize risk to participants.

### Diagnostic methods

RNA extraction was performed using the Hamilton Microlab Star automated system and the Quick-DNA/RNA Viral MagBead kit (Ref. R2141, Zymo Research). After extraction, detection of the E gene was carried using the primers/probe sets described in the Berlin Charité protocol [37] and human ribonuclease P gene (RP) was detected as an internal control [38] using the script enzyme XLT 1-Step RT-qPCR ToughMix L-ROX Kit (Ref. 95134-02 K, Quanta). No-template controls, positive template controls and human specimen controls were included in all runs. Real-time PCR (RT-PCR) was performed using the Applied Biosystems 7500 Fast Real-Time PCR system.

### Patient selection

A total of 3723 patients with positive RT-PCR for SARS-CoV-2 were assessed. From this initial sample, we excluded the cases that had no attached patient notification form or that had an RNAse P control Ct value outside the accepted range of 20 to 35 [11]. We reviewed each patient’s notification form and transcribed the variables of interest into an integrated database, namely: date, name, gender, age, symptomatology, past medical history, and hospital admission. Ct values obtained for each patient were also included. Samples not including complete patient information on symptoms or past medical history were excluded from the study. Patient data were collected as primary data for one registry.

### Statistical analysis

A descriptive analysis of the sociodemographic and epidemiological variables was performed. The quantitative variables were summarized in terms of medians and interquartile range (IQR), and the qualitative variables were summarized in frequencies and proportions. For all continuous values, normality hypotheses were evaluated using the Shapiro-Wilk test. Since a non-normal distribution was obtained, the non-parametric Mann-Whitney-Wilcoxon test was performed to explore potential differences in Ct values between groups. Additionally, for the comparison of more than two groups, the Kruskal-Wallis non-parametric test was used. Tukey’s test was used as a post-hoc test. Likewise, a Spearman correlation and simple linear regression analysis were also performed to calculate the coefficient of determination (R2) to identify relationships between continuous variables. Statistical analyzes were carried out using the R software (RStudio Team 2019). All tests of significance were two-tailed, and P-values < 0.05 were considered statistically significant.

## Results

### Baseline characteristics and association to RT-PCR cycle threshold

A total of 2775 patients were included in the study. The distribution of sex categories, symptomatology, comorbidities, hospitalization status, and median Ct values across the cohort is presented in Table 1. The mean Ct value across the entire sample was 22.7 (range from 10 to 40), with a standard deviation of 5.3 and a median of 22.3.

**Table 1:** Baseline demographic characteristics, symptoms, comorbidities and hospitalization status in 2775 patients infected with SARS-CoV-2

Variables	Categories	Number of patients	Percentage of total	Ct Median
Sex				
	Female	1304	46.9	22.5
	Male	1471	53	22.5
Symptomatology				
	Asymptomatic	764	27.5	22.4
	Cough	1586	57.2	22.2
	Fever	1045	37.6	22.3
	Odynophagia	445	16	21.9
	Respiratory distress	595	21.4	23.2
	Fatigue	1044	37.6	22.2
	Rhinorrhea	28	1	24.6
	Headache	72	2.5	25.5
	Conjunctivitis	2	0.07	21
	Diarrhea	22	0.79	25.7
Prior medical history				
	None	2347	83.7	22.2
	Asthma	39	1.74	21.7
	Chronic obstructive pulmonary disease	96	3.14	23.2
	Diabetes	145	5.67	22.7
	Human immunodeficiency virus infection	8	0.28	24
	Cardiac disease	83	2.89	22.5
	Cancer	11	0.43	22.3
	Malnutrition	6	0.26	24.9
	Obesity	106	3.93	22.3
	Renal failure	33	1.17	22
	Use of immunosuppressants	3	0.1	23
	Smoker	47	1.63	23.8
	Tuberculosis	5	0.18	17.7
Hospitalization				
	Hospitalized	435	15.6	23.5
	Outpatient	2337	84.2	22.1
	No data	3	0.1	22.5

**Fig. 1:**
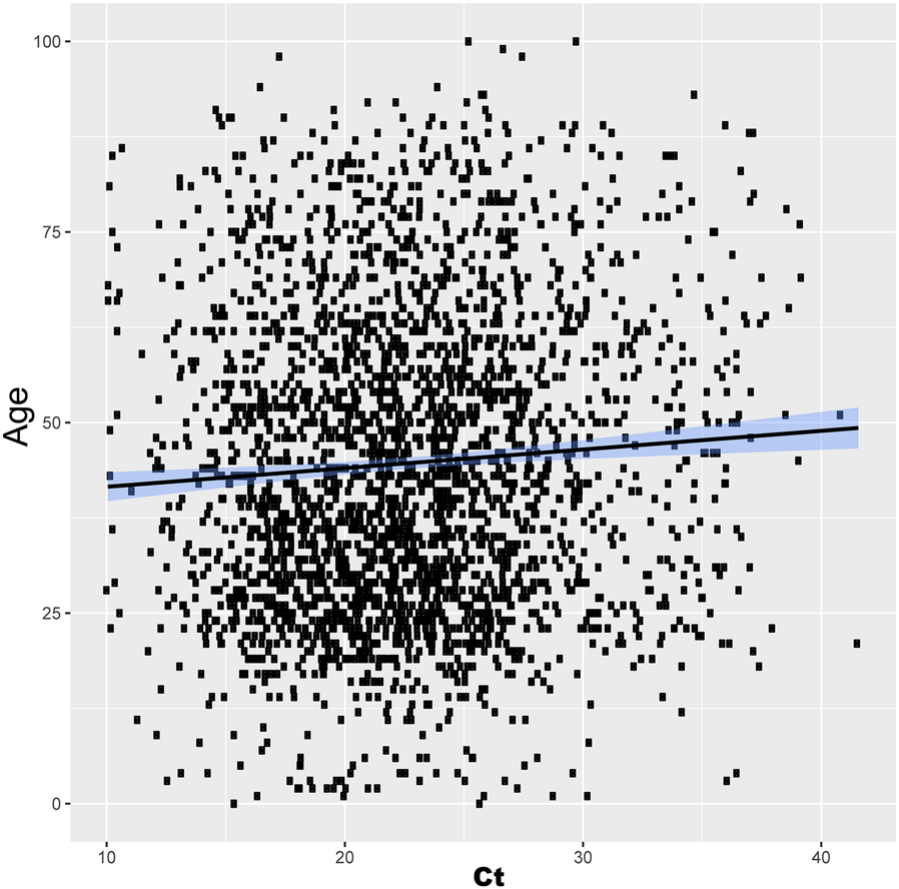
Linear correlation of viral load measured through Cycle threshold (Ct) with age. This analysis was performed in 2775 COVID-19 patients. Although a significant correlation is present (P = 0.0) significant data dispersion is observed

The total study group consisted of 1304 (53 %) males and 1471 (46.9 %) females. The median Ct values between both groups were not significantly different (p = 0.15) at 22.1 and 22.5, respectively (IQR of 6.8 for females and 7.3 for males). The mean age across the study group was 44 years old, ranging from 0 to 100, with a standard deviation of 19 years. Although a significant linear correlation between age and cycle threshold was noticed (p = 0.009), the coefficient of determination was close to zero, and significant data dispersion was present (Fig. 1).

### Prior medical history and association to RT-PCR cycle threshold

**Fig. 2:**
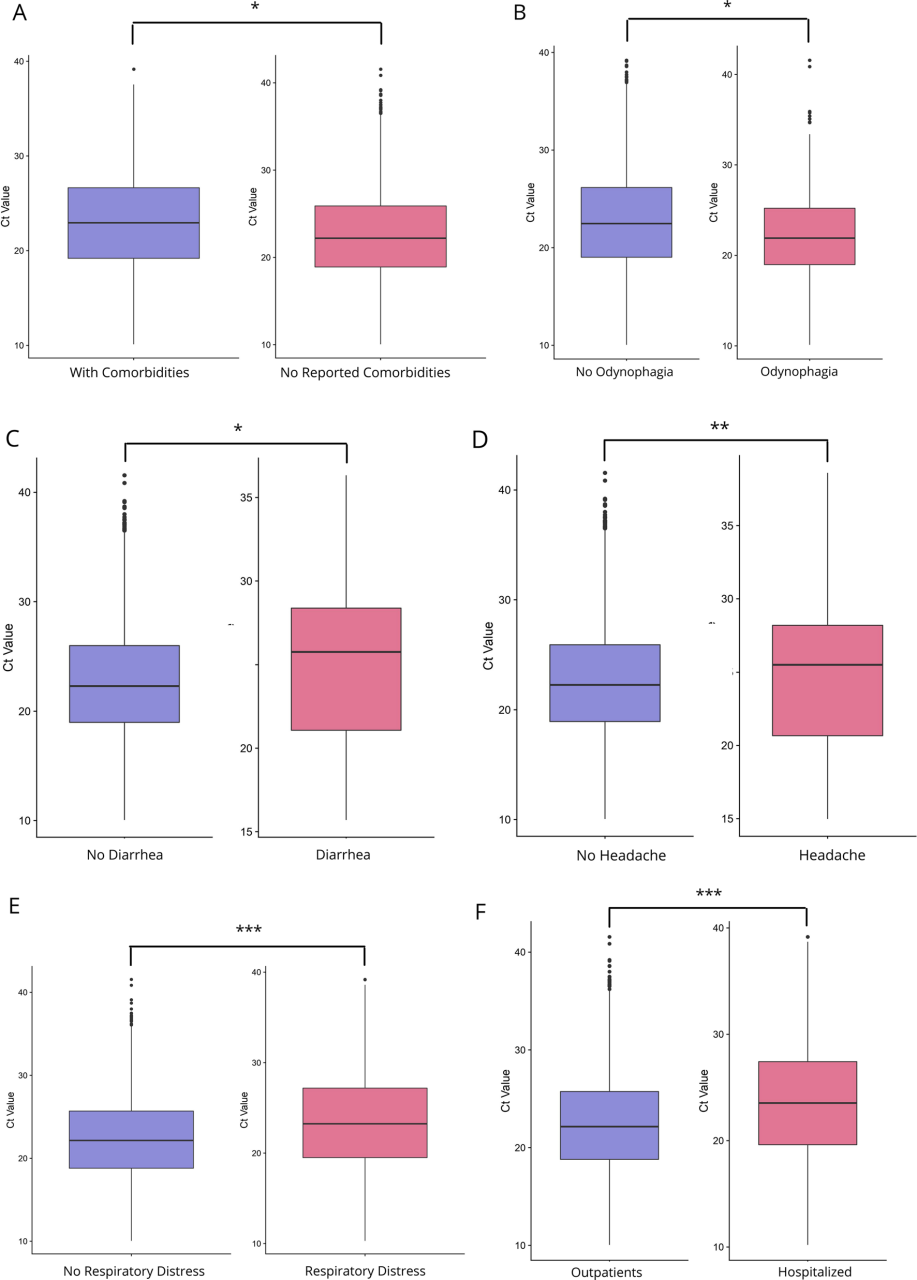
Associations of Ct values with different clinical variables. Shown are box plot histograms representing the associations of RT-PCR Cycle threshold (Ct) values with different clinical variables in patients with COVID-19. Horizonal lines represent medians. P values are included above each box plot comparison. **A** Patients without reported comorbidities have more RNA copies. **B** Patients with odynophagia have lower Ct values than patients without odynophagia. **C** Significant association of between high Ct values (Fewer RNA copies) and diarrhea. **D** Significant association between high Ct values (Fewer RNA copies) and headache. **E** Significant association between high Ct values (Fewer RNA copies) and respiratory distress (F) Significant association between high Ct values (Fewer RNA copies) and hospitalization requirement. *p < 0.05 **p < 0.01 ***p < 0.001* N* no difference

Of the 2775 patients, 2347 (84.5 %) had no relevant medical history. The most common comorbidity, present in 5.2 % of cases (n = 145), was diabetes, followed by obesity (n = 106) and Chronic Obstructive Pulmonary Disease (n = 96). The median Ct of patients with and without comorbidities was 22.2 and 22.9, respectively. This trend on the presence of higher viral RNA in patients without prior medical history was confirmed to be significant (p = 0.02) (Fig. 2A). Among those patients with at least one comorbid condition, a tendency for lower Ct values was observed in patients with tuberculosis (Ct median 17.7; n = 5) and asthma (Ct median 21.78; n = 39); whereas higher values were reported in patients with chronic obstructive pulmonary disease (Ct median 23.2), HIV infection (Ct median 24), and malnutrition (Ct median 24.9). However, no significant correlations could be demonstrated between RNA copies and specific comorbidities (Fig. 2A).

### Clinical characteristics and association to RT-PCR cycle threshold

Of the 2775 patients, 764 (27.5 %) were asymptomatic at the moment of care. Among those who were symptomatic, the most common complaint was cough in 57.2 % of cases (n = 1589), followed by fever (n = 1045) and fatigue (n = 1044) (Table 1). No significant differences were detected between Ct values of symptomatic and asymptomatic patients with Ct medians. Of 22.3 and 22.4, respectively. We noticed three significant correlations between specific symptoms and cycle thresholds: First, patients with odynophagia had more RNA copies than those without (Ct median of 21.9 vs. 22.4, p = 0.03) (Fig. 2B). Conversely, patients with diarrhea (Ct median of 25.7 vs. 22.3, p = 0.04) and headache (Ct median of 25.5 vs. 22.2, p = 0.0008) were found to have higher cycle thresholds (Fig. 2C, D).

We analyzed two markers of disease severity: respiratory distress and hospital admission. Respiratory distress was present in 21.4 % of cases (n = 595), and hospital admission in 15.6 % (n = 435) of the total sample. Patients with respiratory distress tended to have higher Ct values, and a significant association (p = 0.000001) was proven in further analysis (Fig. 2E). Finally, Ct values were significantly higher (p < 0.00007) in hospitalized cases (n = 435) compared to outpatients (n = 2337), with median Ct values of 23.5 and 22.1, respectively (Fig. 2F).

## Discussion

As with other international series, our study found a significant association between Ct values and age [23, 24, 26, 35]. Nevertheless, noticeable data dispersion and a coefficient of determination close to zero challenge this correlation’s meaningfulness (Fig. 1). A potential reason for the weak association is that our sample is, on average, significantly younger than other international cohorts. Thus, the identification of viral load dynamics in elders may be hampered by the relative deficiency of this population in our sample. As a reference, the Magleby cohort’s median age for the high, medium, and low viral load groups was 72, 69, and 63 years, respectively [23]. In comparison, our median sample age was 43 years.

Another finding of our study is that there are no significant differences in Ct values between symptomatic and asymptomatic patients (p = 0.8). This contrasts with what is observed in other viral infections, such as influenza, where symptomatic patients carry up to 100-fold more viral copies than their asymptomatic counterparts [39]. Although other studies report that asymptomatic patients hosted lower RNA copies than their symptomatic counterparts [24], extensive published literature contradicts this claim [8, 15–20]. A recently published study performed in the Colombian territory supports our findings and reports comparable levels of RNA copies between symptomatic and asymptomatic cases [40]. The relevance of these findings is two-fold. First, it indirectly supports the role of the immune responses as the lead driver for symptom development in COVID-19 patients [12, 41]. Second, it adds further evidence to the role of asymptomatic carriers in the spread of SARS-CoV-2. Previous Colombian cohorts have reported frequencies of asymptomatic carriers of 0.91 % in Cundinamarca [42]. Our study suggests this number could be more than twenty-fold higher and coupled with evidence of comparable RNA copies to symptomatic cases. It further highlights the importance of asymptomatic patients in the transmission of SARS-CoV-2.

Regarding the presentation of specific symptomatology, our study detected symptom frequencies that differ from those reported in recent literature (44–46,50). First, the headache was only observed in 2.5 % of patients (n = 72), a finding that contrasts with other cohorts, which show five-fold greater frequencies [43, 44]. Most notably, a report of over 370.000 patients in the United States showed that 34 % of the evaluated patients presented headaches [45]. We also found that patients with headaches had significantly lower RNA copies than those presenting without headache (Fig. 2D). To the best of our knowledge, this is the first study of its kind to report such association. Other cohorts have described how COVID-19 patients with neurological manifestations have undetectable RNA copies in cerebrospinal fluid [46, 47]. This, along with our findings, suggests that patients with COVID-19 probably develop neurological manifestations through indirect mechanisms, such as immune-mediated inflammation and injury [47, 48]. Studies that test for immune reactivity directly from the CSF of COVID-19 patients without neurological symptoms would help support or disprove this hypothesis [48].

We also show a shallow frequency of diarrhea (0.7 %) which contrasts with data from other meta-analyses that report pooled frequencies from 11 to 19 % [45, 49, 50]. Additionally, we also demonstrated that patients with diarrhea had significantly lower RNA copies than patients without gastrointestinal symptoms (Fig. 2C). Besides, the sample source might be critical when interpreting these findings, as other studies have reported high RNA copies in the fecal matter even when upper respiratory samples test negative. For instance, a meta-analysis of 12 studies, including 138 patients, found that 70 % of cases with positive stool samples had negative RT-PCR tests for SARS-CoV-2 [49]. Future studies aimed to evaluate cycle threshold values in upper respiratory and fecal samples in patients with varying degrees of COVID-19 severity across time would help to elucidate the essence of these findings.

Concerning upper respiratory symptomatology, our sample closely resembled the frequencies reported in other large international cohorts [45], with cough present in 57 % of cases (n = 1186) and sore throat in 16 % (n = 445). We also demonstrated a significant correlation between odynophagia and cycle threshold, revealing that patients with sore throat had significantly higher RNA copies levels than patients without it (Fig. 2B). The rationale behind this finding also remains speculative and has not been reported in other international series. However, as it occurs with influenza, it would suggest that the development of odynophagia is a direct effect of viral replication and tissue cytotoxicity [51].

Our study also found that patients with markers of disease severity, namely, respiratory distress and hospital admission, were associated with lower RNA copies (Fig. 2E, F). Although this is supported by other international series [15, 20, 21, 34, 52], evidence from a wide variety of studies contradicts these findings (17,22–32,45). Most notably, Maltezou et al. showed that among 1122 symptomatic and asymptomatic patients in Greece, those with Ct values lower than 25 tended to be at greater risk of intubation and death than patients with Ct values higher than 25 [24]. The study from Magleby et al. replicated these findings with a sample of 678 hospitalized patients in New York [23]. Future large-scale studies should address the potential significance of Ct values in predicting disease severity.

As highlighted by other authors [34, 36], timing since disease onset must be acknowledged as well. Data of temporal profiles of SARS-CoV-2 show that the number of RNA copies peaks close to symptom onset and tapers steeply by the end of the first week [9, 21, 22]. Thus, finding different Ct values in infected patients may tell more about the disease’s timing than the severity itself [34]. Several of the cohorts that show an association between high RNA copies and disease severity either fail to account for timing since disease onset or explicitly declare that patients with more RNA copies also have shorter periods from disease onset to sampling [24, 26, 53]. Furthermore, and considering the sizeable proportion of COVID-19 patients that show clinical deterioration to severe disease after a week of symptoms, this might explain why some studies, like ours, report an inverse association between RNA copies and disease severity [15, 21, 34].

Finally, the present study can serve as a point of reference for future Colombian cohorts that aim to study similar variables to the ones shown here and assess the relevance of the newly found SARS-CoV-2 variants inside Colombian territory [54, 55]. In particular, three characteristics make our cohort a helpful point of comparison. First, our cohort is homogeneous, as it was obtained only from the department of Cundinamarca with the exclusion of Bogotá. Second, it was restricted to the first pandemic wave in Colombia. Furthermore, third, recent evidence [55, 56] has proven that during the timeline of our study (March-July 2020), four SARS-CoV-2 lineages were detected in the Cundinamarca department (A.1, B.1, B.1.153, B.1.420). Future lines of research that examine the repercussions of newly identified SARS-CoV-2 variants [54] should use this cohort as a baseline to compare and more strongly determine the impact of the new variants on clinical outcomes. This is especially important considering the recent evidence that links the circulation of two variants of concern in the country (B.1.1.7 and P.1)[54, 55], with improved fitness advantage, increased transmissibility and viral loads [57–63].

In conclusion, our study represents the first large-scale Colombian cohort composed of symptomatic, asymptomatic, hospitalized, and ambulatory patients correlating RNA copies with demographic and clinical variables. We demonstrated that patients with no prior medical history had higher RNA copies than patients with comorbidities. We also found that no significant cycle threshold differences existed between patients with or without symptoms, highlighting the importance of asymptomatic carriers in the spread of SARS-CoV-2. Among symptomatic patients, we observed strong correlations between RNA copies and odynophagia, diarrhea, and headache. Finally, an inverse relationship between RNA copies and markers of disease severity is demonstrated. A limitation to our current study is the lack of controls for confounding variables such as timing. Furthermore, we did not count with longitudinal data to differentiate asymptomatic from presymptomatic patients. Because of this, further research that controls for confounding variables not assessed in our cohort should be conducted to clarify the nature of these associations and shed light on the Ct value’s actual utility in evaluating and managing patients with COVID-19. Future research done with patients from Cundinamarca can use this study as a point of comparison to better elucidate the impact that new SARS-CoV-2 variants have on RNA copies, symptoms and outcomes in Colombian patients.

## Supplementary Information

**Additional file 1: Figure S1.** Geographic location of Cundinamarca, Colombia Colombian territory is shown. Department of Cundinamarca is highlighted.

**Additional file 2: Figure S2. **Colombian Standardized SARS-CoV-2 Report Form. The demographic and clinical data of patients with suspected SARS-CoV-2 infection must be reported to the Ministry of Health through this form.

## Data Availability

All data generated or analyzed during this study are included in this published article and its supplementary information files (S1 Appendix).
