# Supplementary figures and images for: Contrasting SARS-CoV-2 RNA copies and clinical symptoms in a large cohort of Colombian patients during the first wave of the COVID-19 pandemic
Source: Ann Clin Microbiol Antimicrob. 2021 May 24;20:39. doi: 10.1186/s12941-021-00445-8 (PMC8142070; doi:10.1186/s12941-021-00445-8)

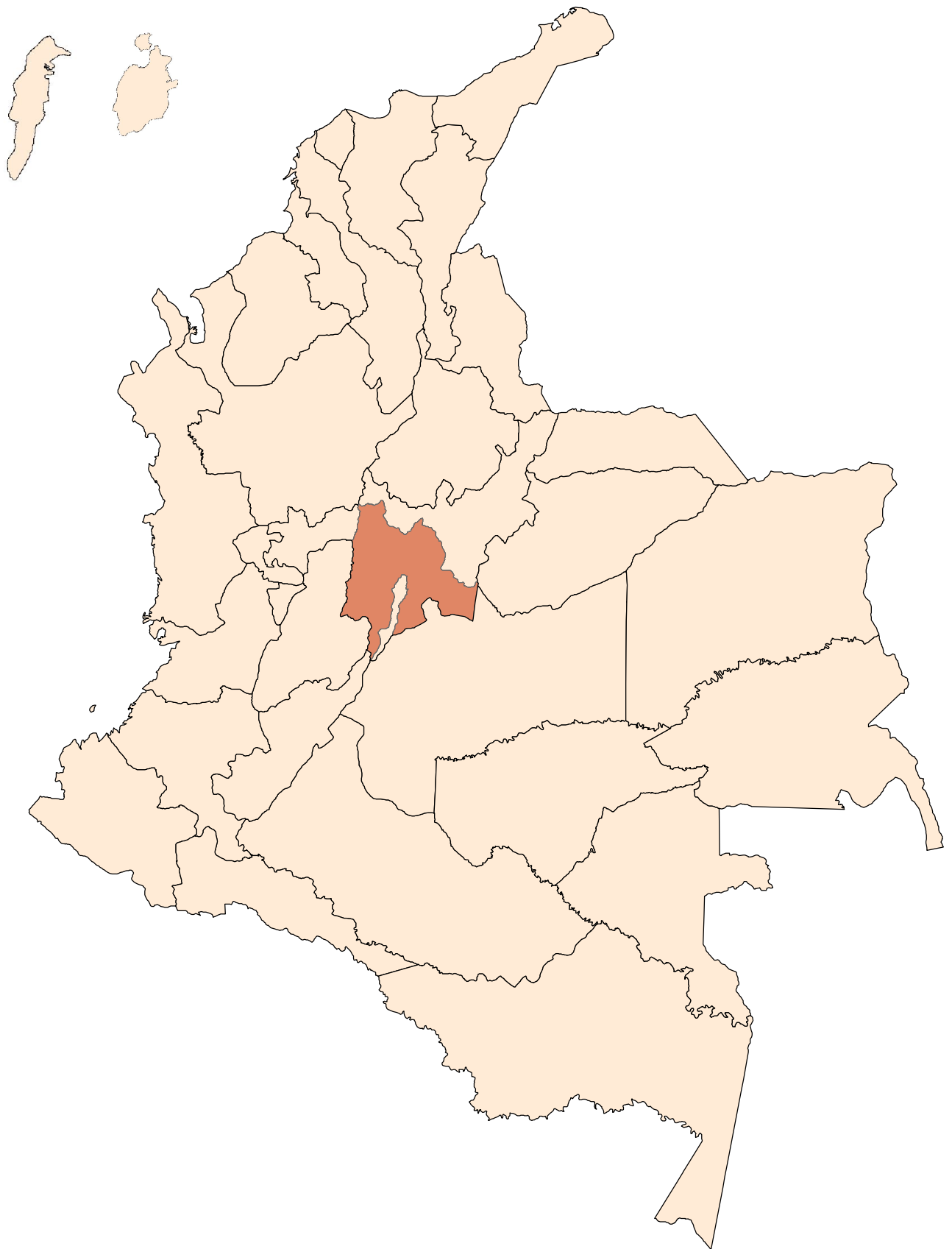

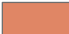 Cundinamarca

0 50 100 200 300 400  
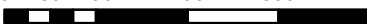 Kilometers

Supplement: Supplementary file 1 — Additional file 1: Figure S1. Geographic location of Cundinamarca, Colombia Colombian territory is shown. Department of Cundinamarca is highlighted. [file 12941_2021_445_MOESM1_ESM.pdf]
