# Supplementary material for: Contrasting SARS-CoV-2 RNA copies and clinical symptoms in a large cohort of Colombian patients during the first wave of the COVID-19 pandemic
Source: Ann Clin Microbiol Antimicrob. 2021 May 24;20:39. doi: 10.1186/s12941-021-00445-8 (PMC8142070; doi:10.1186/s12941-021-00445-8)
Supplement: Supplementary file 2 — Additional file 2: Figure S2. Colombian Standardized SARS-CoV-2 Report Form. The demographic and clinical data of patients with suspected SARS-CoV-2 infection must be reported to the Ministry of Health through this form. [file 12941_2021_445_MOESM2_ESM.pdf]

INSTITUTO NACIONAL DE SALUD

SISTEMA NACIONAL DE VIGILANCIA EN SALUD PÚBLICA
Subsistema de información SIVIGILA
Ficha de notificación individual

Datos básicos

La ficha de notificación es para fines de vigilancia en salud pública y todas las entidades que participen en el proceso deben garantizar la confidencialidad de la información LEY 1273/09 y 1266/09

1. INFORMACIÓN GENERAL

FOR-R02.0000-001 V:09 2020-03-06

1.1 Código de la UPGD

Departamento | Municipio | Código | Sub– Índice

Razón social de la unidad primaria generadora del dato

1.2 Nombre del evento

Código del evento

1.3 Fecha de la notificación (dd/mm/aaaa) __ / __ / ____

2. IDENTIFICACIÓN DEL PACIENTE

2.1 Tipo de documento

○ RC ○ TI ○ CC ○ CE ○ PA ○ MS ○ AS ○ PE ○ CN

2.2 Número de identificación

*RC : REGISTRO CIVIL | TI : TARJETA IDENTIDAD | CC : CÉDULA CIUDADANÍA | CE : CÉDULA EXTRANJERÍA |- PA : PASAPORTE | MS : MENOR SIN ID | AS : ADULTO SIN ID | PE : PERMISO ESPECIAL DE PERMANENCIA | CN : CERTIFICADO DE NACIDO VIVO

2.3 Nombres y apellidos del paciente

2.4 Teléfono

2.5 Fecha de nacimiento (dd/mm/aaaa) __ / __ / ____

2.6 Edad

2.7 Unidad de medida de la edad

○ 1. Años ○ 3. Días ○ 5. Minutos
○ 2. Meses ○ 4. Horas ○ 0. No aplica

2.8 Sexo

○ M. Masculino ○ I. Indeterminado
○ F. Femenino

2.9 Nacionalidad

2.10 País de ocurrencia del caso

Código

2.11 Departamento y municipio de procedencia/ocurrencia

Departamento | Municipio

2.12 Área de ocurrencia del caso

○ 1. Cabecera municipal ○ 3. Rural disperso
○ 2. Centro poblado

2.13 Localidad de ocurrencia del caso

2.14 Barrio de ocurrencia del caso

Código

2.15 Cabecera municipal/centro poblado/rural disperso

2.16 Vereda/zona

2.17 Ocupación del paciente

Código

2.18 Tipo de régimen en salud

○ P. Excepción ○ C. Contributivo ○ N. No Asegurado
○ E. Especial ○ S. Subsidiado ○ I. Indeterminado/ pendiente

2.19 Nombre de la administradora de Planes de beneficios

Código

2.20 Pertenencia étnica

○ 1. Indígena Grupo ètnico ______ ○ 2. Rom, Gitano ○ 3. Raizal ○ 4. Palenquero ○ 5. Negro, mulato afro colombiano ○ 6. Otro

2.21 Estrato

2.22 Seleccione los grupos poblacionales a los que pertenece el paciente

❑ Discapacitados ❑ Migrantes ❑ Gestantes Sem.de gestaciòn ❑ Población infantil a cargo del ICBF ❑ Desmovilizados ❑ Víctimas de violencia armada
❑ Desplazados ❑ Carcelarios ❑ Indigentes ❑ Madres comunitarias ❑ Centros psiquiátricos ❑ Otros grupos poblacionales

3. NOTIFICACIÓN

3.1 Fuente

○ 1. Notificación rutinaria ○ 4. Búsqueda activa com.
○ 2. Búsqueda activa Inst. ○ 5. Investigaciones
○ 3. Vigilancia Intensificada

3.2 País, departamento y municipio de residencia del paciente

País | Departamento | Municipio

3.3 Dirección de residencia

3.4 Fecha de consulta (dd/mm/aaaa) __ / __ / ____

3.5 Fecha de inicio de síntomas (dd/mm/aaaa) __ / __ / ____

3.6 Clasificación inicial de caso

○ 1. Sospechoso ○ 3. Conf. por laboratorio
○ 2. Probable ○ 4. Conf. Clínica
○ 5. Conf. nexo epidemiológico

3.7 Hospitalizado

○ Sí ○ No

3.8 Fecha de hospitalización (dd/mm/aaaa) __ / __ / ____

3.9 Condición final

○ 1. Vivo
○ 2. Muerto
○ 0. No sabe, no responde

3.10 Fecha de defunción (dd/mm/aaaa) __ / __ / ____

3.11 Número certificado de defunción

3.12 Causa básica de muerte

3.13 Nombre del profesional que diligenció la ficha

3.14 Teléfono

4. ESPACIO EXCLUSIVO PARA USO DE LOS ENTES TERRITORIALES

4.1 Seguimiento y clasificación final del caso

○ 0. No aplica ○ 4. Conf. Clínica ○ 6. Descartado ○ D. Descartado por error de digitación
○ 3. Conf. por laboratorio ○ 5. Conf. nexo epidemiológico ○ 7. Otra actualización

4.2 Fecha de ajuste (dd/mm/aaaa) __ / __ / ____

Correos: sivigila@ins.gov.co

SISTEMA NACIONAL DE VIGILANCIA EN SALUD PÚBLICA
Subsistema de información SIVIGILA
Ficha de notificación individual – Datos complementarios

INSTITUTO NACIONAL DE SALUD

REPÚBLICA DE COLOMBIA

Infección respiratoria aguda por virus nuevo. Cod INS 346

La ficha de notificación es para fines de vigilancia en salud pública y todas las entidades que participen en el proceso deben garantizar la confidencialidad de la información LEY 1273/09 y 1266/09

EVENTO DE NOTIFICACIÓN INMEDIATA

RELACIÓN CON DATOS BÁSICOS FOR-R02.0000-075 V:01 2020-05-08

A. Nombres y apellidos del paciente	B. Tipo de ID*	C. Número de identificación

*RC : REGISTRO CIVIL | TI : TARJETA IDENTIDAD | CC : CÉDULA CIUDADANÍA | CE : CÉDULA EXTRANJERÍA |- PA : PASAPORTE | MS : MENOR SIN ID | AS : ADULTO SIN ID | PE : PERMISO ESPECIAL DE PERMANENCIA | CN : CERTIFICADO DE NACIDO VIVO

5. ¿POR QUÉ SE NOTIFICA EL CASO COMO IRA POR VIRUS NUEVO?

5.1 ¿Viajó a áreas de circulación del virus? ○ 1. Sí ○ 2. No

5.1.1¿El viaje fue en el territorio nacional? ○ 1. Sí ○ 2. No

5.1.1.1¿Dónde? ______ Departamento/Municipio

5.1.2 ¿El viaje fue Internacional? ○ 1. Sí ○ 2. No

5.1.2.1 ¿Dónde? ______ Código País

5.2 ¿Tuvo contacto estrecho en los últimos 14 días con un caso probable o confirmado con infección respiratoria aguda grave por virus nuevo? ○ 1. Sí ○ 2. No

5.3 ¿Reporta alguno de los siguientes síntomas?

❑ Tos ❑ Fiebre ❑ Dolor de garganta (Odinofagia) ❑ Dificultad respiratoria ❑ Fatiga o adinamia ❑ Rinorrea ❑ Conjuntivitis

❑ Cefalea ❑ Diarrea ❑ Pérdida de olfato y/o gusto ❑ Otros

5.3.1 ¿Cuáles otros? ______

6. ANTECEDENTES CLÍNICOS

6.1 ¿Reporta alguno de los siguientes antecedentes clínicos?

❑ Asma ❑ EPOC ❑ Diabetes ❑ VIH ❑ Enfermedad cardiaca ❑ Cancer ❑ Malnutrición ❑ Obesidad ❑ Insuficiencia renal

❑ Toma medicamentos inmunosupresores ❑ Fumador ❑ Hipertensión ❑ Tuberculosis ❑ Otros

6.1.1 ¿Cuáles otros? ______

7. DIAGNÓSTICO Y TRATAMIENTO

7.1 Si se tomó de radiografía de tórax ¿Qué hallazgos se presentaron? ○ 1. Infiltrado alveolar o neumonía ○ 2. Infiltrados intersticiales ○ 4. Infiltrados basales en vidrio esmerilado ○ 3. Ninguno

7.2 Servicio en el que se hospitalizó ○ 1. Hospitalización general ○ 3. UCI

7.2.1 Fecha de ingreso a UCI (dd/mm/aaaa) ☐☐-☐☐-☐☐☐☐

7.3 Si hubo complicaciones, ¿Cuáles se presentaron?

❑ 1. Derrame pleural
❑ 2. Derrame pericárdico
❑ 3. Miocarditis
❑ 4. Septicemia
❑ 5. Falla respiratoria
❑ 6. Otro

7.3.1 Otros cuáles? ______

8. DATOS DE LABORATORIO

La información relacionada con laboratorios debe ingresarse a través del módulo de laboratorios del aplicativo sivigila

Tome 3 a 5 C.C. de sangre en tubo seco y una muestra para identificación viral (hisopado nasofaríngeo, aspirado nasofaríngeo, aspirado bronquial)

	Fecha de toma (dd/mm/aaaa)	Fecha de recepción (dd/mm/aaaa)	Muestra	Prueba	Agente	Resultado	Fecha de recepción (dd/mm/aaaa)	Valor registrado
9.1								
9.2								

Marque así		
	**Muestra**	1. Sangre total | 3. Hisopado nasofaríngeo | 4. Tejido | 8. Aspirado nasofaríngeo | 11. Otros líquidos esteriles | 22. Lavado bronquial
	**Prueba**	2. IgM | 3. IgG | 4. PCR | 30. Patología | 31. Inmunohistoquímica | 46. Inhibición hemaglutinación | 55. Cultivo | 76. IFI | 92. Hemocultivo | E1. Aislamiento viral | F3. Determinación de antígeno | H9. IgG - IgM
	**Agente**	8. Otro | 16. Adenovirus |18. Virus sincitial respiratorio | 22. Haemophilus influenzae | 24. Streptococcus pneumoniae | 40. Influenza A | 41. Influenza | 42. Parainfluenza 1 | 43. Parainfluenza 2 | 44. Parainfluenza 3 | 56. Enterovirus| 59. Influenza A(H1N1) pdm09 | 64. Influenza A no subtipificable | 76. Bocavirus | 77. Coronavirus | 78. Metaneumovirus | 79. Rinovirus | 84. Virus respiratorios |1Q. Coronavirus causante del síndrome respiratorio de Oriente Medio (MERS-CoV) | 1R. Coronavirus subtipo 229e| 1S. Coronavirus subtipo HKU1 | 1T. Coronavirus subtipo NL63 | 1U. Coronavirus subtipo OC43 | 1V. Influenza A(H3N2) | 1W. Parainfluenza tipo 4 | 2H. Coronavirus subtipo COVID19
	**Resultado**	1. Positivo | 2. Negativo | 3. No procesado | 4. Inadecuado | 6. Valor registrado | 12. Contaminado con hongos | 13. Muestra escasa de células |

Correo: sivigila@ins.gov.co

INSTRUCTIVO DILIGENCIAMIENTO FICHAS DE NOTIFICACIÓN
DATOS BÁSICOS

Definición
La ficha de notificación de Datos Básicos es un instrumento que permite obtener **información prioritaria** de las **variables de tiempo, lugar y persona** para la notificación de los diferentes eventos de interés en salud pública, con el fin de diferenciar claramente las características propias de las poblaciones que se ven asociadas a los eventos notificados (variables universales), los lugares y momentos específicos en los que se presentan.

Consideraciones generales
Para el diligenciamiento de la ficha de datos básicos, es importante tener en cuenta los aspectos que se numerarán a continuación, con el fin que la información allí consignada cumpla con los criterios de calidad y veracidad del dato:
1. Diligencie con letra imprenta, clara y legible; recuerde que la ficha de notificación es un **DOCUMENTO MÉDICO LEGAL**, por lo tanto es necesario que **evite enmendaduras o tachones** durante su diligenciamiento.
2. Utilice preferiblemente esfero de tinta negra para el diligenciamiento de la ficha de notificación.
3. La ficha debe ser diligenciada en su totalidad.
4. Tenga en cuenta que dependiendo la variable puede o no tener múltiples opciones de respuesta. Por lo anterior, las variables cuyas categorías estén designadas con un (círculo) significa que esa variable tiene una **ÚNICA** opción de respuesta. Por el contrario las variables que están señaladas con un (cuadrado), significa que tiene **MÚLTIPLES** opciones de respuesta.
5. Escriba una letra o número por casilla.
6. Verifique que las variables Código de evento y nombre del evento, coincidan.
7. Verifique que las variables código de UPGD, número de identificación, tipo de documento y código del evento, estén diligenciadas correctamente, con el fin de evitar contratiempos en la notificación.
8. Recuerde que cuando el evento requiere datos complementarios es necesario diligenciar también la ficha de datos específicos "Cara B", la cual encontrará en la página web del INS, con el nombre del evento.
Excepciones: en los eventos Bajo peso al nacer y Muerte perinatal y neonatal tardía, los datos básicos corresponden a los datos de la madre.

1. INFORMACIÓN GENERAL			
**VARIABLE**	**CATEGORÍAS Y DEFINICIÓN**	**CRITERIOS SISTEMATIZACIÓN**	**OBLIG**
**1.1 Código de UPGD**	Dilligencie el código de habilitación del prestador.<br>Tenga en cuenta:<br>**Departamento:** se relaciona con el código del departamento al que pertenece la institución (código DIVIPOLA del DANE) y corresponde a los dos primeros dígitos.<br>**Municipio:** los tres dígitos sumados a los anteriores, identifican el municipio donde se encuentra ubicada la institución y corresponden al código DIVIPOLA del DANE.<br>**Código:** el primer dígito de este grupo, corresponde al número asignado cuando la institución se encuentra en el registro de habilitación de prestadores de salud (REPS). Tenga en cuenta que si la UPGD pertenece a la Policía Nacional el dígito debe ser 7, si pertenece a fuerzas militares debe ser 8. Si son unidades informadoras que no tienen código de habilitación debe ser 9. Los cuatro dígitos siguientes son asignados por el departamento-municipio cuando son unidades informadoras; de lo contrario son asignados por el REPS.<br>**Sub-índice:** identifica el número de sede al que pertence la institución cuando la UPGD tiene más de una (la sede principal siempre es 01). Cuando terminan en 80, se relacionan con UPGD que pertencen al ejército nacional; 81, a la Armada; 82, a la fuerza aérea; 83, hospital militar; 55 EAPB y 99, identifican los establecimientos penitenciarios y carcelarios.	Registre el código de habilitación por el módulo de caracterización de UPGD antes de iniciar la notificación.<br>Recuerde clasificar la UPGD como Activa = Si, en el módulo de caracterización de UPGD.	**SI**
**1.2 Nombre del evento**	Nombre del evento y código de acuerdo al listado de clasificación establecido por el Instituto Nacional de Salud.	* Tener en cuenta que el nombre y el código del evento deben coincidir para evitar confusiones durante la sistematización.	**SI**
**1.3 Fecha de notificación**	Fecha en la que se está informando el evento al siguiente nivel por cualquier mecanismo, en especial para los eventos de notificación inmediata. Formato día-mes-año	* La fecha de notificación debe ser mayor o igual a la fecha de inicio de síntomas y a la fecha de consulta.	**SI**
**2. IDENTIFICACIÓN DEL PACIENTE**			
**2.1 Tipo de documento**	Se relaciona con el tipo de documento de identidad que tiene el paciente que está siendo notificado. Diligencie una sola opción en esta variable.<br>Tenga en cuenta que si el paciente es menor de edad y no tiene identificación debera ingresarlo como "MS" y el número de identificación debera seguir las indicaciones del apartado 2.2.<br>En caso que el paciente sea mayor de edad y no tiene identificación debera ingresarlo como "AS" y el número de identificación debera seguir las indicaciones del apartado 2.2.	* El tipo de documento debe ser consistente con la edad del paciente.	**SI**
**2.2 Número de identificación**	El estandar de longitud de caracteres del número de identificación acorde al tipo de identificación, se define en el "Diccionario de elemento de datos el estandar" lenguaje comun de intercambio de información (MinTIC), de la siguiente manera:<br><br>RC: entre 8 ó 10 ú 11 dígitos<br>TI: 10 y 11 dígitos<br>CC: entre 8 y 17 dígitos<br>CE: menor a 11 dígitos<br>PA: menor a 19 dígitos<br>Menor sin identificación: para este último ingrese el documento de la madre con el consecutivo respectivo al número de nacimiento.<br>Mayor sin identificación: para mayores de 18 años utilice el código del Departamento + código de Municipio y el consecutivo definido por el Entidad Territorial o UPGD.<br>PE: menor a 18 dígitos<br>CN: 9 dígitos<br><br>En adopción de la Circular Externa 000029 del 16 de agosto de 2017 expedida por el Ministerio de Salud y Protección Social, se incluye validación para la identificación de extranjeros de acuerdo al estándar internacional ISO 3166-1 para los siguientes tipo de documento: en CE , PA, AS, MS, de esta manera se aumenta la longitud de captura en 3 dígitos más para cada uno.	Diligencie la variable, de lo contrario el sistema no permitirá continuar con el ingreso de la información.	**SI**
**2.3 Nombres y apellidos del paciente**	Corresponde a los nombres y apellidos del paciente textual al documento de identificación.	* El primer nombre y primer apellido son obligatorios para el sistema, de lo contrario no le permitirá continuar con la sistematización de la información.	**SI**
**2.4 Teléfono**	Corresponde al número telefónico (fijo o celular) donde pueda ubicarse al paciente. Es necesario anteceder el número indicativo del departamento o distrito, ejemplo, para marcar a Bogotá, (1) 2302162.<br><br>Tenga en cuenta que el diligenciamiento correcto de esta variable permitira contactar al paciente en caso de ser requerido.	* Si no es posible obtener el número telefónico, diligencie esta variable "SIN INFORMACIÓN". De lo contrario se tomará arbitrariamente durante la sistematización de la ficha.	**SI**
**2.5 Fecha de nacimiento**	Para el evento de Bajo peso al nacer y mortalidad perinatal los datos en la ficha de datos básicos corresponden a los de la mama.<br>Formato día-mes-año.	El ingreso de la fecha de nacimiento permitirá el cálculo de la semana epidemiológica para Sífilis congénita y Síndrome de rubeola congénita.	**NO**
**2.6 Edad**	Reporte la edad cumplida. Si se desconoce indague por la fecha de nacimiento.	El sistema calcula la edad si se tiene la fecha de nacimiento.	**SI**
**2.7 Unidad de medida de la edad**	Cantidad estandarizada en la que se mide el tiempo que ha vivido una persona desde el nacimiento.	* En los casos donde el tipo de identificación sea CC, TI o CE se toma la unidad de medida 1.<br>* Tenga en cuenta que la variable debe coincidir con el tipo de identificación del paciente, de lo contrario el sistema no permitirá continuar con el ingreso de la información.<br>La unidad de medida 0="no aplica" solo puede ser utilizada para el evento 215 defectos congénitos, cuando el diagnóstico se realiza prenatal	**SI**

INSTRUCTIVO DILIGENCIAMIENTO FICHAS DE NOTIFICACIÓN
DATOS BÁSICOS

**2.8 Sexo**	Se relaciona con el sexo del paciente relacionado con la notificación.<br>M = Masculino<br>**F =** Femenino<br>**I =** Indetermiando<br>Los eventos Mortalidad Perinatal y Bajo Peso al Nacer capturan datos de la madre.<br>El sexo I=Indeterminado aplica únicamente para Defectos Congénitos.	* Tener en cuenta que al sistematizar la información se digita la variable TAL CUAL aparece en la ficha de notificación, debido a que no existe una regla de validacion que permita validarla con el nombre del paciente.	**SI**
**2.9 Nacionalidad**	Corresponde al estado o nación que pertenece una persona según su documento de identificación.	Seleccione el país correspondiente a la nacionalidad según la lista establecida.	**SI**
**2.10 País de ocurrencia del caso**	Indage al caso o familiar del mismo, respecto al país donde ocurrió la exposición al evento de interés en salud pública a notificar.<br>Tenga en cuenta que la ocurrencia o procedencia corresponde al lugar geográfico donde posiblemente el paciente adquirio o al cual se atribuye la exposición al agente o factor de riesgo que ocasiono el evento. En los eventos transmisibles se deberan considerar los tiempos de incubación de la enfermedad.	* Tener en cuenta el diligenciamiento correcto de esta variable en la ficha, debido a que su ausencia se tomaría por defecto en el sistema como "país donde ocurre el caso : Colombia"	**SI**
**2.11 Departamento y municipio de procedencia/ocurrencia**	Ingrese el nombre del departamento y municipio de donde proviene/ocurre el caso.<br>Tenga en cuenta que la ocurrencia o procedencia corresponde al lugar geográfico donde posiblemente el paciente adquirio o al cual se atribuye la exposición al agente o factor de riesgo que ocasiono el evento. En los eventos transmisibles se deberan considerar los tiempos de incubación de la enfermedad.	Revisar que el Municipio coincida con el Departamento.<br>Recuerde que pueden haber coincidencias en el nombre del Municipio en diferentes Departamentos.	**SI**
**2.12 Área de ocurrencia del caso**	**1. Cabecera Municipal:** es el área geográfica que está definida por un perímetro urbano, cuyos límites se establecen por acuerdos del Concejo Municipal. Corresponde al lugar en donde se ubica la sede administrativa de un municipio.<br>**2. Centro Poblado:** Se define como una concentración de mínimo veinte (20) viviendas contiguas, vecinas o adosadas entre sí, ubicada en el área rural de un municipio o de un Corregimiento Departamental.<br>**3. Rural Diperso:** es el área que se caracteriza por la disposición dispersa de viviendas y explotaciones agropecuarias. No cuenta con un trazado o nomenclatura de calles, carreteras, avenidas y demás.<br>*Nota: tenga en cuenta los criterios para definir la ocurrencia de un evento descritos en el numeral 2.10.*	* Variable de única selección. No se puede ingresar al sistema más de una categoría.	**SI**
**2.13 Localidad de ocurrencia**	Ingrese el nombre de la localidad donde ocurrió el caso.<br>Tenga en cuenta que esta variable aplica para los Distritos como Bogotá, Municipios como Medellín y Cali, entre otros; los cuales están sectorizados internamente según el departamento de planeación municipal.<br>*Nota: tenga en cuenta los criterios para definir la ocurrencia de un evento descritos en el numeral 2.10.*	* Depende de la categoría seleccionada en la variable 2.11 "cabecera municipal"	**SI**
**2.14 Barrio de ocurrencia del caso**	Ingrese el nombre del barrio donde ocurrió el caso.<br>*Nota: tenga en cuenta los criterios para definir la ocurrencia de un evento descritos en el numeral 2.10.*	* Depende de la categoría seleccionada en la variable 2.11 "cabecera municipal". La codificación depende de la tabla de barrios definidos por la entidad territorial.	**SI**
**2.15 Cabecera municipal/centro poblado/rural disperso**	Nombre del área de ocurrencia del caso seleccionado en la variable 2.11<br>*Nota: tenga en cuenta los criterios para definir la ocurrencia de un evento descritos en el numeral 2.10.*	* Depende de la categoría seleccionada en la variable 2.11	**SI**
**2.16 Vereda/zona**	Ingrese el nombre de la vereda o zona donde ocurrió el caso.<br>*Nota: tenga en cuenta los criterios para definir la ocurrencia de un evento descritos en el numeral 2.10.*	* Depende de la categoría seleccionada en la variable 2.11 "rural disperso"	**SI**
**2.17 Ocupación del paciente**	Describa claramente la ocupación principal de paciente (en el caso que aplique).	* La codificación de la ocupación está a partir de la Clasificación Internacional Uniforme de Ocupaciones (CIUO 88)	**SI**
**2.18 Tipo de régimen en salud**	Se relaciona con el régimen de afiliación al sistema general de seguridad social en salud, en el que se encuentra el caso que está siendo notificado o su acudiente.<br>Variable de única respuesta.<br>**P= Excepción**: régimen de seguridad social de los afiliados al Fondo Nacional de prestaciones Sociales del magisterio, de servidores públicos de Ecopetrol así como los servidores públicos de universidades públicas<br>**C= Contributivo:** sistema de salud mediante el cual todas las personas vinculadas a través de contrato de trabajo, los servidores públicos, los pensionados y jubilados y los trabajadores independientes con capacidad de pago, hacen un aporte mensual (cotización) al sistema de salud.<br>**E= Especial:** regímenes de seguridad social de los afiliados a la Policía Nacional, Fuerzas Militares, Armada, Fuerza Aérea.<br>**S= Subsidiado:** El Régimen Subsidiado es el mecanismo mediante el cual la población más pobre, sin capacidad de pago, tiene acceso a los servicios de salud a través de un subsidio que ofrece el Estado.<br>**N= No asegurado**<br>**I= Indeterminado/ pendiente**: esta opción debe ser utilizada<br>exclusivamente para ajuste por las EAPB, cuando el caso no<br>corresponde a esta y requiere verificación en el nivel nacional	* Para la sistematización de la información, se toma una sola categoría de respuesta.	**SI**
**2.19 Nombre de la administradora de salud**	Ingrese el nombre de la empresa que presta los servicios de salud al caso la cual deberá ser acorde al tipo de régimen en salud.	* Para la sistematización de la información se tiene en cuenta el nombre de la administradora que debe coincidir con la categoría seleccionada en la variable 2.17	**SI**
**2.20 Pertenencia étnica**	"La pertenencia étnica se define como patrimonio cultural compartido por un grupo de personas", las definiciones para cada uno de los grupos étnicos son*:<br>*Indígena* : persona de ascendencia amerindia que comparten sentimientos de identificación con su pasado aborigen, manteniendo rasgos y valores propios de su cultura tradicional, así como formas de organización y control social propios. Si diligencia esta opción deberá elegir el grupo étnico al que pertenece.<br>*Rom, Gitano:* Son comunidades que tienen una identidad étnica y cultural propia; se caracterizan por una tradición nómada, y tienen su propio idioma que es el romanés.<br>*Raizal:* Población ubicada en el Archipiélago de San Andrés, Providencia y Santa Catalina, con raíces culturales afro-anglo-antillanas, cuyos integrantes tienen rasgos socioculturales y lingüísticos claramente diferenciados del resto de la población afrocolombiana.<br>*Palenquero:* Población hubicada en el municipio de San Basilio de Palenque, departamento de Bolívar, donde se habla el palenquero, lenguaje criollo.<br>*Negro o afrocolombiano:* persona de ascendencia afrocolombiana que poseen una cultura propia, y tienen sus propias tradiciones y costumbre dentro de la relación campo-poblado.<br><br>Tenga en cuenta que esta variable se diligencia con base a lo que el paciente manifiesta, mas no a la percepción de quien está diligenciando la ficha.	* Para la sistematización de la información se tiene en cuenta la categoría única diligenciada en la variable.<br><br>Variable con categoría única de respuesta.	**SI**
**2.21 Estrato**	Estrao socio-económico donde esta ubicada la vivienda del paciente, estos son:<br>1. Bajo-bajo, 2. Bajo, 3. Medio-bajo, 4. Medio, 5. Medio-alto o 6. Alto	Variable con categoría única de respuesta.	**SI**
**2.22 Seleccione los grupos poblacionales a los que pertenece el paciente**	La variable tiene múltiples opciones de respuesta que se pueden diligenciar dependiendo de lo referido por el paciente o de lo observado por quien esté diligenciando la ficha de notificación, a continuación se definen algunas pobalciones relevantes para tener mayor claridad:<br><br>*Migrante* : persona que se desplaza o se ha desplazado a través de una frontera internacional o dentro de un país, fuera de su lugar habitual de residencia independientemente de: 1) su situación jurídica; 2) el carácter voluntario o involuntario del desplazamiento; 3) las causas del desplazamiento; o 4) la duración de su estancia (OIM).<br>*Desmovilizado:* Aquel que por decisión individual abandone voluntariamente sus actividades como miembro de organizaciones armadas al margen de la ley, esto es grupos guerrilleros y de autodefensa, y se entregue a las autoridades de la República (Decreto 128 de 2003).<br>*Desplazados* : Personas o grupos de personas que han sido forzadas u obligadas a abandonar sus hogares o lugares de residencia habitual, en particular como resultado de o para evitar los efectos del conflicto armado, situaciones de violencia generalizada, violaciones de derechos humanos o desastres naturales o causados por el hombre, y que no han cruzado fronteras reconocidas internacionalmente" (Naciones Unidas, documento E/CN.4/1992/23).<br><br>Si no pertenece a algún grupo poblacional, se sugiere señalar la opción "otros grupos poblacionales".	* Para la sistematización de la información se hace necesario marcar la/s categorías de respuesta necesarias según corresponda. Si no pertenece a algún grupo se toma como "otros grupos poblacionales"	**SI**

INSTRUCTIVO DILIGENCIAMIENTO FICHAS DE NOTIFICACIÓN
DATOS BÁSICOS

**3. NOTIFICACIÓN**			
**3.1 Fuente**	Corresponde a la fuente de la notificación lo cual influye en el análisis de datos y en las acciones de vigilancia y control, las posibles fuentes son: 1. notificación rutinaria, 2. Búsqueda Activa Institucional, 3. Vigilancia intesificada, 4. Búsqueda Activa Comunitaria y 5. Investigaciones		
**3.2 País, departamento y municipio de residencia del paciente**	Nombre del departamento y municipio donde reside el paciente al momento de la notificación.<br>Nota: *Para definir residencia acogemos el concepto de la Ley 1607 de 2012 que establece que una persona es residente de algun lugar geográfico si "permanece continua o discontinuamente en el país por más de ciento ochenta y tres (183) días calendario incluyendo dias de entrada y salida.*		**SI**
**3.3 Dirección de residencia**	Dirección donde se encuentra ubicada la residencia del paciente al momento de la notificación acorde a los parámetros establecidos en el numeral 3.2		**SI**
**3.4 Fecha de consulta**	Formato día/mes/año. Se diligencia la fecha en la que consultó el paciente por el evento notificado.	* Para sistematizar la información tenga en cuenta que la fecha de consulta NO puede ser mayor a la fecha de notificación. Si esto sucede se tomará como error en el sistema y no se podrá continuar con la digitación de la ficha de notificación.	**SI**
**3.5 Fecha de inicio de síntomas**	Formato día/mes/año. Se diligencia la fecha del primer día en que el paciente inició síntomas del evento acorde a la definición de caso.	* Para sistematizar la información tenga en cuenta que la fecha de inicio de síntomas NO debe ser superior a la fecha de consulta y a la fecha de notificación. Si esto sucede se tomará como un error en el sistema y no se podrá continuar con la digitación de la ficha de notificación.	**SI**
**3.6 Clasificación inicial del caso**	En la notificación inicial, seleccione únicamente una categoría de la variable.<br>Cada evento tiene una clasificación de caso específica, la cual es acorde a lo descrito en el protocolo.<br>Es importante que aquellos eventos que ingresan probables y sospechosos, sean ingresados con esa clasificación ya que se entendera que son detectados de forma oportuna, de lo contrario, es decir, si ingresan confirmados, corresponden a una falla en la pronta identificación del evento.	*Para la sistematización, los valores permitidos en esta variable dependen del evento que se va a notificar.	**SI**
**3.7 Hospitalizado**	Marque con una X la opción según corresponda.<br>Hospitalización debida al evento que se está notificando.	* Ingrese la información solicitada, de lo contrario el sistema no permitirá continuar con el ingreso.	**SI**
**3.8 Fecha de hospitalización**	Formato día/mes/año. Se diligencia la fecha en la que el paciente fue hospitalizado como consecuencia del evento que se está notificando.	* Tenga en cuenta que para la sistematización la fecha de hospitalización no puede ser inferior a la fecha de consulta, ni a la fecha de notificación.	**SI**
**3.9 Condición final**	Variable de única selección.<br>0 = Solo aplica cuando se capte el caso por BAI y se desconoce el dato, o para defectos congénitos cuando la unidad de medida es "0"<br>1 = Vivo<br>2 = Muerto<br>Tenga en cuenta que es importante establecer si la muerte fue debida al evento de interés en salud pública para diligenciar esta variable.	* Tenga en cuenta que para la sistematización de la información se debe diligenciar la condición final del paciente al momento de la notificación, la cual se derive del evento que se está notificando.	**SI**
**3.10 Fecha de defunción**	Formato día/mes/año. Se diligencia la fecha en la que el paciente fallece como consecuencia del evento que se esta notificando. Se debe diligenciar si en la variable "3.8 Condición final" es 2 = Muerto"	* Se diligencia dependiendo de la respuesta de la variable 3.8 "2 = Muerto".	**SI**
**3.11 Número de certificado de defunción**	Número consecutivo que aparece en la parte superior derecha del certificado de defunción diligenciado si la "3.8 Condición final" es 2 = Muerto"	* Se diligencia dependiendo de la respuesta de la variable 3.8 "2 = Muerto". Debe estar diligenciado el registro, de lo contrario el sistema no permitirá contiuar con la sistematización de la ficha.	**SI**
**3.12 Causa básica de muerte**	Diagnóstico CIE X, que ocasionó la muerte del caso y que esta relacionado con el evento de interés en salud pública.	* Se diligencia una vez la respuesta de la variable 3.8 sea "2 = Muerto"	**SI**
**3.13 Nombre de profesional que diligencia la ficha**	Nombre de la persona que diligenció la ficha de notificación en la UPGD o UI	* Para la sistematización de la información debe diligenciar los datos solicitados, de lo contrario el sistema no permitirá el ingreso de la ficha de notificación.	**SI**
**3.14 Teléfono**	Número telefónico de contacto de la persona que realiza la notificación, preferiblemente el número celular para poder contactarlo en caso de ser requerido.	* Para la sistematización de la información debe diligenciar los datos solicitados, de lo contrario el sistema no permitirá el ingreso de la ficha de notificación.	**SI**
**4. ESPACIO EXCLUSIVO PARA USO DE LOS ENTES TERRITORIALES**			
**4.1 Seguimiento y clasificación final del caso**	Estos son los valores que se relacionan para definir la clasificación final del caso, posterior de obtener resultados de laboratorio o análisis del caso.<br>Corresponde a la clasificación definitiva del caso.<br>0 = No aplica (es el valor por defecto del sistema si el caso es nuevo o no ha sido ajustado)<br>3 = Confirmado por laboratorio<br>4 = Confirmado por clínica<br>5 = Confirmado por nexo epidemiológico<br>6= Descartado: puede ser por criterio epidemiológico, por laboratorio, porque no cumple la condición del caso, etc.<br>7 = Otra actualización: quiere decir que se puede modificar cualquier campo excepto código de evento, Upgd, numero de id, fecha de notificación. Esto, después de haber notificado el caso.<br>D = Error de digitación por las siguientes razones:<br>- el evento notificado es errado, por ejemplo, se digitaron los datos para el evento parotiditis y realmente se trataba de un caso de varicela.<br>- cuando al menos una de las variables de la llave primaria (código de evento, año, semana epidemiológica, código de prestador, código sub-índice del prestador, tipo de identificación y número de identificación) fueron mal digitadas, ya que es posible modificarlas una vez hayan sido notificadas (generación del archivo plano).	Los tipos de ajuste permitos y los tiempos para el ajuste de casos, se encuentran definidos en los protocolos de vigilancia en salud pública.	**SI**
**4.2 Fecha de ajuste**	Fomato día/mes/año; corresponde a la fecha en la que se ajustó la clasificación inicial del caso.		**SI**

INSTRUCTIVO DILIGENCIAMIENTO FICHAS DE NOTIFICACIÓN DATOS COMPLEMENTARIOS
Infección respiratoria aguda por virus nuevo Cod INS: 346

VARIABLE	CATEGORÍAS Y DEFINICIÓN	CRITERIOS SISTEMATIZACIÓN	OBLIG
**A. Nombres y apellidos del paciente**<br>**B. Tipo de ID**<br>**C. Número de identificación**	Es necesario diligenciar estos datos en la ficha de complementarios, los cuales debe coincidir con la información registrada en la ficha de datos básicos.	* El tipo de documento debe ser coincidente con la edad del paciente.<br>* El tipo y número de documento debe coincidir con la información ingresada en la ficha de datos básicos.	**SI**
**5. ¿POR QUÉ SE NOTIFICA EL CASO COMO UN IRA POR VIRUS NUEVO?**			
**5.1 ¿Viajó a áreas de circulación del virus?**	Marque SI o NO según corresponda<br>Para el diligenciamiento de la variable tenga en cuenta que estos criterios catalogan al caso que se está notificando como un **caso de IRA por virus nuevo. Si marca la opción "Viajó areas de circulacion del virus"** diligencie las variables según corresponda.	Diligencie la variable, de lo contrario el sistema no le permitirá continuar con el ingreso de la información.	**SI**
**5.1.1 ¿El viaje fue en el territorio Nacional? 5.1.1.1 ¿Donde?**	Marque SI o NO según corresponda<br>1. Sí 2.No<br>Escriba código de departamento y municipio	Diligencie la variable, de lo contrario el sistema no le permitirá continuar con el ingreso de la información.	**SI**
**5.1.2 ¿El viaje fue internacional?**<br>**5.1.2.1 ¿Donde?**	Marque SI o NO según corresponda<br>1. Sí 2.No<br>Escriba código de país	Diligencie la variable, de lo contrario el sistema no le permitirá continuar con el ingreso de la información.	**SI**
**5.2 ¿Tuvo contacto estrecho en los últimos 14 días con un caso probable o confirmado con infección respiratoria aguda grave por virus nuevo?**	Marque SI o NO según corresponda	Diligencie la variable, de lo contrario el sistema no le permitirá continuar con el ingreso de la información.	**SI**
**5.3 Síntomas 5.3.1 Cuales otros?**	Marque con una X la opción según corresponda	Diligencie la variable, de lo contrario el sistema no le permitirá continuar con el ingreso de la información.	**SI**
**6. ANTECEDENTES CLINICOS**			
**6.1 ¿Reporta alguno de los siguientes antecedentes clínicos? 6.1.1. Cuales otros?**	Marque con una X la opción según corresponda	Diligencie la variable, de lo contrario el sistema no le permitirá continuar con el ingreso de la información.	**SI**
**7. DIAGNÓSTICO Y TRATAMIENTO**			
**7.1 Si se tomó de radiografia de tórax ¿Qué hallazgos se presentaron?**	Marque con una X la opción según corresponda.<br>1 = Inflíltrado alveolar o neumonía<br>2 = Infiltrados intersticiales<br>4 = Infiltrados basales en vidrio esmerilado<br>3 = Ninguno	Diligencie la variable, de lo contrario el sistema no le permitirá continuar con el ingreso de la información.	**SI**
**7.2 Servicio en el que se hospitalizó**	Marque con una x la opción según corresponda.<br>1 = Hospitalización general<br>3 = UCI	Diligencie la variable, de lo contrario el sistema no le permitirá continuar con el ingreso de la información.	**SI**
**7.2.1 Fecha de ingreso a UCI**	Diligencie la fecha con formato (dd/mm/aaaa)	Diligencie la variable, de lo contrario el sistema no le permitirá continuar con el ingreso de la información.	**SI**
**7.5 Si hubo complicaciones ¿cuáles se presentaron?**	Marque con una X la opción según coprresponda. Si selecciona la categoría de "otras", mencione cuáles.<br>1. Derrame pleural: Acumulación anormal de liquido en el espacio pleural.<br>2. Derrame pericárdico acumulación anormal de líquido en la cavidad pericárdica.<br>3. Miocarditis: inflamación del tejido miocardico.<br>4. Septicemia: infección sistémica causada por un foco infeccioso inicial.<br>5. Falla respiratoria:alteración de la homeostasis respiratoria, que afecta el recambio gaseoso pulmonar.<br>6.Otros	Diligencie la variable, de lo contrario el sistema no le permitirá continuar con el ingreso de la información. En las complicaciones que presente el paciente diligencie la opción 1 = Si; de lo contrario marque la opción 2 = No	**SI**
**8. DATOS DE LABORATORIO (Ingrese los datos en el módulo de laboratorio correspondiente en el Sivigila)**			
**8.1 - 8.2 Fecha de toma**<br>**Fecha de reccepción**<br>**Muestra**<br>**Prueba**<br>**Agente**<br>**Resultado**<br>**Fecha de recepción**<br>**Valor registrado**	Diligencie los criterios a partir de las siguientes opciones:<br>**MUESTRAS**: 1. Sangre total | 3. Hisopado nasofaríngeo | 4. Tejido | 8. Aspirado nasofaríngeo | 11. Otros líquidos esteriles | 22. Lavado bronquial<br>**PRUEBA**: 2. IgM | 3. IgG | 4. PCR | 30. Patología | 31. Inmunohistoquímica | 46. Inhibición hemaglutinación | 55. Cultivo | 76. IFI | 92. Hemocultivo | E1. Aislamiento viral | F3. Determinación de antígeno | H9. IgG - IgM<br>**AGENTE**:8. Otro | 16. Adenovirus |18. Virus sincitial respiratorio | 22. Haemophilus influenzae | 24. Streptococcus pneumoniae | 40. Influenza A | 41. Influenza | 42. Parainfluenza 1 | 43. Parainfluenza 2 | 44. Parainfluenza 3 | 56. Enterovirus| 59. Influenza A(H1N1) pdm09 | 64. Influenza A no subtipificable | 76. Bocavirus | 77. Coronavirus | 78. Metaneumovirus | 79. Rinovirus | 84. Virus respiratorios |1Q. Coronavirus causante del síndrome respiratorio de Oriente Medio (MERS-CoV) | 1R. Coronavirus subtipo 229e| 1S. Coronavirus subtipo HKU1 | 1T. Coronavirus subtipo NL63 | 1U. Coronavirus subtipo OC43 | 1V. Influenza A(H3N2) | 1W. Parainfluenza tipo 4 | 2H. Coronavirus subtipo COVID19<br>**RESULTADOS**: 1. Positivo | 2. Negativo | 3. No procesado | 4. Inadecuado | 6. Valor registrado | 12. Contaminado con hongos | 13. Muestra escasa de células |		
